# The Effect of Educational Intervention on Knowledge, Attitude, and Practices Regarding Colorectal Cancer Screening Practices Among Nursing Students in Jordan

**DOI:** 10.7759/cureus.66111

**Published:** 2024-08-04

**Authors:** Majdi M Alzoubi, Suhair Al-Ghabeesh, Batool Qutami

**Affiliations:** 1 Department of Clinical Nursing, Al-Zaytoonah University, Amman, JOR; 2 Department of Emergency Medicine, Specialty Hospital, Amman, JOR

**Keywords:** practices, attitude, knowledge, screening, health education, colorectal cancer

## Abstract

Background: Colorectal cancer (CRC) is the second leading cause of cancer-related deaths worldwide. This study aimed to examine the impact of an educational intervention on nurses' knowledge, attitude, and practice regarding CRC screening in Jordan.

Methodology: The study was an experimental design conducted before and after the educational intervention. Participants attended four 30-minute sessions held weekly over one month. A paired t-test was used to determine the mean difference in participants' knowledge, attitude, and practice regarding CRC screening before and after the intervention. Statistical analyses were conducted using IBM SPSS Statistics for Windows, Version 27.0 (IBM Corp., Armonk, NY).

Results: A total of 43 participants took part in the study, of whom 29 (67.4%) were female and 14 (32.6%) were male. The majority (30, 69.8%) were aged between 20 and 30 years. The results showed a significant mean increase in knowledge (mean difference [MD] = 3.09, *P* < 0.001), attitude (MD = 4.16, *P* < 0.001), and practice (MD = 2.67, *P* < 0.001) regarding CRC screening following the intervention.

Conclusions: The implementation of health education about CRC screening for the study participants was successful based on the results. This strategy could provide a solid basis for organizing, implementing, and supervising CRC screening initiatives.

## Introduction

Over the years, colorectal cancer (CRC) has become the third most commonly diagnosed cancer and the second leading cause of cancer-related mortality worldwide [1,2]. Several studies indicate that advanced screening tools are now available for detecting and reducing the occurrence of CRC [2,3]. Despite this, the incidence of CRC cases is rising, with estimates predicting 2.2 million cases and approximately 1.1 million deaths globally by 2030 [1]. The incidence of CRC is particularly increasing among individuals under 50 in developed countries, including the United States, Canada, the United Kingdom, France, Australia, New Zealand, and Japan [4-8]. This rise in incidence among younger individuals may be attributed to lifestyle factors such as diet and obesity [9].

CRC is the second most common type of cancer in both men and women in Jordan, making up 11.3% of all new cases [10]. The overall age-standardized rate was 16.3 per 100,000, with males and females at 15.9 and 16.6 per 100,000, respectively, among the Jordanian population [10]. The causes of Jordan's high incidence of CRC are not yet fully understood. Recently, however, the Jordanian Ministry of Health has begun to focus more on CRC as a health issue that has to be resolved by informing the populace about the disease's prevention, screening, and early identification.

According to the World Health Organization (WHO), 40% of cancers are preventable, and 40% are curable if detected early [11]. Jordan has less population knowledge than is necessary to promote the growth of screening behavior of CRC [12]. Fecal immunochemical testing (FIT), flexible sigmoidoscopy every 10 years with an FIT test every year, or a colonoscopy every 10 years are the best screening methods for CRC [13]. These methods will gradually extend the number of years that these CRC patients can live with a high quality of life, but they will also necessitate more colonoscopies to be conducted in the community [13,14].

Understanding the state of a population in terms of their present knowledge levels, practices, and attitudes toward cancer prevention is a crucial aspect in determining cancer control initiatives. The overall five-year and 10-year survival rates for CRC in Jordan were 58.2% and 51.8%, respectively. Low survival rates were associated with older age, poor differentiation, advanced cancer stage, and right-sided tumors [10]. However, CRC is one of the cancers that can be largely prevented through early detection. Given this, the knowledge gap among nursing students must therefore be understood for teachers to improve their teaching methods and student learning outcomes [15]. The inclusion of cancer prevention in students’ curricula has been proposed by medical educators to improve long-term retention of knowledge, positive self-reported attitudes and beliefs, and an intent to apply prevention in future practice [16]. Hence, this study aimed to determine whether an educational intervention for nursing students would increase their knowledge, attitudes, and practice, as well as increase patients’ participation in CRC screening.

The degree to which people are aware of CRC and screening procedures, as well as their opinions on these topics, affect CRC screening. Student nurse practitioners may have a significant impact on raising screening rates in the community's asymptomatic high-risk populations through education, particularly for those who operate in primary care offices and community clinics [17]. The likelihood of screening and early detection of CRC cases in Jordan may rise as a result of the knowledge gained through research examining whether group education interventions influence awareness and attitudes towards CRC. This knowledge is important since nurses are crucial to preventative healthcare initiatives in primary care settings. Additionally, there were no previous studies conducted to determine the effect of a health education intervention on knowledge, attitude, and perceived barriers regarding CRC risk factors and screening techniques among nursing students in Jordan.

## Materials and methods

Study design

An experimental study was conducted to determine the effect of educational intervention on the participant’s knowledge, attitude, and practices toward CRC screening. The target population of this study was nursing students in Jordan. The participant received four 30-minute sessions held weekly over one month. The participants completed the study questionnaires before and after the educational intervention.

Sample size

The sample size was estimated using the G Power program (version 3.0.10, Heinrich-Heine-Universität Düsseldorf, Düsseldorf, Germany) for this study. The parameters used were 80% power, medium effect size of 0.50, and a significance level of α = 0.05 (two-tailed). The estimated sample size was 34 participants. Because of the expected attrition rate, a 20% dropout rate was added, making the adjusted sample size to be 43. The study adopted a nonprobability convenience sampling method to recruit nursing students who met the inclusion criteria for the study.

Ethical consolidation

This study was approved by the Institutional Review Board of Al-Zaytoonah University (reference no. 2022-2023/17/29), written informed consent was obtained, and the purpose of the study was explained to all participants. Patients' confidentiality was protected. All data were available only to the authors.

Intervention

The relevant studies in the evidence-based literature were used to create the health education intervention's content [18-21]. The educational intervention is divided into four sections according to the participants, who are Jordanians at average risk for CRC. Basic information about CRC is included in the first section, including its definition, pathogenesis, symptoms, and incidence in Jordan. The purpose of this section was to improve participants' understanding and perceptions of their susceptibility to CRC. To raise participants' perceptions of their vulnerability to CRC and the severity of the disease, the risk factors for CRC are presented in the second section. The final section offers potential CRC screening choices to raise participants' perceptions of the benefits of screening and lower their perceptions of its limitations. In the fourth section, CRC treatment options are discussed, along with survival information for both early and late detection. The effectiveness of the content intended for the study participants was evaluated by experts from community health nursing and oncology who reviewed the educational intervention.

Instrument

The instrument used was the Colorectal Cancer Knowledge, Perceptions, and Screening Survey (CRCKPSS) [22]. The CRCKPSS is composed of three main sections. The first section, titled *The CRC Knowledge Test*, assesses participants' knowledge in three key areas, including CRC incidence and risk, warning signs and symptoms, myths, and truths, and performing screening tests, with an internal consistency of 0.8. In the second section, labeled *Health Perceptions*, participants' perceptions of their susceptibility to CRC, the seriousness of CRC, the advantages of screening, and the obstacles to screening are assessed, with an internal consistency of 0.85. The last section assesses the participants' CRC screening behaviors using nine questions with a yes/no response option [22]. Additionally, the questionnaire consisted of demographic characteristics of the participants, including age, gender, residence, year of study, and marital status.

Statistical analysis

Initially, the preliminary data analysis was performed for data cleaning to investigate missing values and wrong data entry. The statistical analyses employed in the study were descriptive statistics and paired t-tests. The descriptive analysis presents the frequencies, mean, and standard deviation of the study variables. The paired t-test was performed to determine the mean difference in the participants’ knowledge, attitude, and practice regarding CRC screening before and after intervention. The statistical analyses were performed using IBM SPSS Statistics for Windows, Version 27.0 (IBM Corp., Armonk, NY).

## Results

Table 1 summarizes the general characteristics of the study participants. Among the 43 individuals who took part, 29 (67.4%) were female and 14 (32.6%) were male. The majority of participants (30, 69.8%) were aged between 20 and 30 years. The highest educational level achieved by most participants was a bachelor's degree (16, 37.2%). Additionally, 30 (69.8%) participants were single, 18 (41.9%) were employed full-time, 24 (55.8%) were self-supported, and 34 (79.1%) had no family history of CRC.

**Table 1:** General characteristics of the study participants (n = 43).

Variables	F = frequency (%)
Age (Years)	
20-30	30 (69.8)
31-40	8 (18.6)
41-50	5 (11.6)
Gender	
Female	29 (67.4)
Male	14 (32.6)
Education	
Diploma	15 (34.9)
Bachelor	16 (37.2)
Masters	12 (27.9)
Marital status	
Single	30 (69.8)
Married	13 (30.2)
Employment	
Unemployed	13 (30.2)
Self-employed	3 (7.0)
Part time	6 (14.0)
Full time	18 (41.9)
Retired	3 (7.0)
Financial support	
Self-supported	24 (55.8)
Supported by family	16 (37.2)
Supported by government	2 (4.7)
Others	1 (2.3)
Family history	
No	34 (79.1)
Yes	7 (16.3)
I don’t know	2 (4.7)

Table 2 displays the mean differences in knowledge scores for CRC screening before and after intervention. The results reveal a significant mean increase in knowledge score after intervention (MD = 3.09, *P* < 0.001).

**Table 2:** Mean difference of knowledge toward colorectal cancer screening before and after intervention. SD, standard deviation; CI, confidence interval; DF, degree of freedom

Knowledge	Mean (SD)	MD (95% CI)	*t*-stat (DF)	P
Pre-intervention	12.67 (3.54)	3.09 (1.79-4.390)	4.79 (42)	< 0.001
Post-intervention	15.77 (2.07)			

Table 3 displays the mean differences in attitude scores for CRC screening before and after intervention. The results reveal a significant mean increase in attitude score after intervention (MD = 4.16, *P* < 0.001).

**Table 3:** Mean difference of attitudes toward colorectal cancer screening before and after intervention. SD, standard deviation; CI, confidence interval; DF, degree of freedom

Attitude	Mean (SD)	MD (95% CI)	*t*-stat (DF)	P
Pre-intervention	34.07 (4.73)	4.16 (2.41-5.92)	4.78 (42)	<0.001
Post-intervention	38.23 (2.32)			

Table 4 displays the mean differences in practice scores for CRC screening before and after intervention. The results reveal a significant mean increase in practice score after intervention (MD = 2.67, *P* < 0.001).

**Table 4:** Mean difference of practices toward colorectal cancer screening before and after intervention. SD, standard deviation; CI, confidence interval; DF, degree of freedom

Practice	Mean (SD)	MD (95% CI)	*t*-stat (DF)	P
Pre-intervention	2.35 (1.72)	2.67 (1.98-3.37)	7.74 (42)	<0.001
Post-intervention	5.02 (1.52)			

## Discussion

The purpose of this study was to determine how health education on CRC screening can improve nursing students' knowledge, attitudes, and practices regarding CRC screening in Jordan. Given the alarming incidence rate of CRC and the critical role of screening in survival rates, ensuring that people seek medical attention for suspicious symptoms and utilize screening programs is an essential strategy to reduce cancer mortality [8,23]. The lack of educational resources and facilities for participating in CRC screening programs may be the primary reason for people's poor knowledge and participation in these screenings [21,23-25]. Nurses are pivotal in enhancing the effectiveness of CRC screening programs, improving patient outcomes, and contributing to early detection and prevention [26]. Therefore, understanding nurses' knowledge, attitudes, and practices regarding CRC is vital, particularly when providing emotional support and counseling to patients who may be anxious or fearful about screening and potential results.

The present study's findings showed a significant increase in knowledge score after intervention, with a mean increase of 3.09. The findings of a recent study by Rakhshani et al. reveal a significant increase in the experimental group's mean knowledge scores following the intervention, highlighting the importance of education in enhancing participant knowledge [23]. This highlights the essential of doctors, healthcare professionals, and media outlets to advise and encourage individuals to undergo screening tests and participate in training sessions to prevent CRC [27]. Retraining programs for medical staff and community-based interventions can also significantly contribute to reducing the incidence of this disease [28]. Poor knowledge about CRC significantly lowers screening rates [28,29]. Consequently, adequate knowledge is a key factor in promoting the adoption of preventive measures within the study population [28].

The participants' attitudes regarding CRC screening mean scores significantly increased after the intervention, with a mean increase of 4.16. While no prior study specifically examined the impact of educational interventions on participants' attitudes toward CRC screening, a recent study by Rakhshani et al. reported a substantial improvement in CRC screening behavior following a health education intervention [28]. Health professionals assert that understanding the benefits of a habit can facilitate behavior modification [30]. One could argue that individuals with gastrointestinal malignancies are more likely to modify their behavior if they comprehend the personal benefits of doing so [27]. As a result, education promotes positive attitudes about the importance of CRC screening, motivating nurses to actively support screening initiatives.

Furthermore, the participants' practices regarding CRC screening mean scores significantly increased after the intervention, with a mean increase of 2.67. Educational interventions are essential for enhancing nursing practices related to CRC screening [26,28]. By increasing knowledge, improving communication skills, fostering positive attitudes, and boosting self-efficacy, these interventions empower nurses to deliver higher-quality care [28]. Consequently, this leads to better patient outcomes, more effective screening programs, and a reduction in the incidence and mortality of CRC. Similarly, Rakhshani et al. discovered that cues to action regarding CRC screening in their experimental group nearly doubled following the intervention [28].

The study does have some limitations. First, it relies on interview questionnaires, which were inevitably collected through self-reporting and are associated with a response bias. Second, because the data was gathered from a single hospital, it may not be generalizable to the entire community. Therefore, it is recommended to conduct additional research with control and experimental groups, including follow-up periods, to determine the long-term effects of education on CRC screening behaviors in both groups. Additionally, similar research should be conducted in other cities and regions to create a suitable screening environment for the general public.

## Conclusions

The study's overall findings reveal a significant increase in mean knowledge, attitude, and practice toward CRC screening after completing the educational program. This indicates that the implemented training program had a positive impact. Consequently, it can be concluded that the participants greatly benefited from the educational intervention in this study. Therefore, enhancing these factors, such as providing basic information about CRC, including its definition, pathogenesis, symptoms, and incidence, can promote CRC screening behaviors.

Continuing education for this group of patients should be encouraged to prevent subsequent complications and to improve their quality of life. This is the responsibility of all healthcare providers working with those patients.
